# RNA Polymerase II Mutations Conferring Defects in Poly(A) Site Cleavage and Termination in *Saccharomyces cerevisiae*

**DOI:** 10.1534/g3.112.004531

**Published:** 2013-02-01

**Authors:** Charles E. Kubicek, Robert D. Chisholm, Sachiko Takayama, Diane K. Hawley

**Affiliations:** Department of Chemistry and Institute of Molecular Biology, University of Oregon, Eugene, Oregon 97403-1229

**Keywords:** polyadenylation, eukaryotic transcription, *rpb2* gene mutations

## Abstract

Transcription termination by RNA polymerase (Pol) II is an essential but poorly understood process. In eukaryotic nuclei, the 3′ ends of mRNAs are generated by cleavage and polyadenylation, and the same sequence elements that specify that process are required for downstream release of the polymerase from the DNA. Although Pol II is known to bind proteins required for both events, few studies have focused on Pol II mutations as a means to uncover the mechanisms that couple polyadenylation and termination. We performed a genetic screen in the yeast *Saccharomyces cerevisiae* to isolate mutations in the N-terminal half of Rpb2, the second largest Pol II subunit, that conferred either a decreased or increased response to a well-characterized poly(A) site. Most of the mutant alleles encoded substitutions affecting either surface residues or conserved active site amino acids at positions important for termination by other RNA polymerases. Reverse transcription polymerase chain reaction experiments revealed that transcript cleavage at the poly(A) site was impaired in both classes of increased readthrough mutants. Transcription into downstream sequences beyond where termination normally occurs was also probed. Although most of the tested readthrough mutants showed a reduction in termination concomitant with the reduced poly(A) usage, these processes were uncoupled in at least one mutant strain. Several *rpb2* alleles were found to be similar or identical to published mutants associated with defective TFIIF function. Tests of these and additional mutations known to impair Rpb2−TFIIF interactions revealed similar decreased readthrough phenotypes, suggesting that TFIIF may have a role in 3′ end formation and termination.

Programmed transcription termination—the dissociation of RNA polymerase (RNAP) from the DNA template and nascent RNA in response to encoded signals—is necessary to confine elongation complexes to a single transcription unit, prevent interference with downstream gene expression, and recycle the polymerases (reviewed in Gilmour and Fan 2008; Richard and Manley 2009; Peters *et al.* 2011). For bacterial RNAPs, transcription termination also is responsible for creating the 3′ ends of mRNAs. In contrast, the 3′ ends of eukaryotic nuclear mRNAs, which are synthesized by RNA polymerase (Pol) II, are primarily generated by internal cleavage of the nascent transcript, followed by the addition of a poly(A) tail.

Investigation of Pol II termination has shown that polyadenylation and termination are functionally coupled and share required proteins and nucleic acid sequences (reviewed in Bentley 2005; Buratowski 2005). Cleavage and poly(A) addition are directed by positioning and efficiency elements located upstream and downstream of the poly(A) site (reviewed in Zhao *et al.* 1999; Richard and Manley 2009). These same nucleic acid sequences also are required for dissociation of Pol II from the template, which occurs at multiple positions that can be hundreds of base pairs downstream of the poly(A) site.

Two general classes of models have been proposed to explain how 3′ end processing signals are transmitted to Pol II to induce termination. The first, the “antiterminator” or “allosteric” model, proposes that the set of accessory proteins bound to Pol II is changed upon passage of the elongation complex through polyadenylation-specifying sequences (Logan *et al.* 1987). The second model, often called the “torpedo” mechanism, suggests that cleavage of the transcript generates an unprotected (*i.e.*, uncapped) 5′ end, which allows entry of a termination protein (Connelly and Manley 1988).

The two models are not mutually exclusive. Indeed, both have some experimental support, and neither appears sufficient to explain all 3′ end processing and termination events (Buratowski 2005; Luo *et al.* 2006; Richard and Manley 2009). The torpedo model gained support with the discovery of a 5′-3′ exonuclease important to termination in yeast and mammals (Kim *et al.* 2004; West *et al.* 2004). However, experiments *in vitro* have suggested that degradation of the RNA by Rat1, the exonuclease implicated in termination in yeast, may not be sufficient for disassembly of the ternary elongation complex (Dengl and Cramer 2009).

Regardless of the mechanistic details, the models share the common feature that accessory proteins must associate with the nascent RNA, the RNAP, or both to bring about termination. Consistent with that idea, a number of proteins required for both polyadenylation and termination in yeast bind to the C-terminal domain (CTD) of the largest Pol II subunit, Rpb1 (reviewed in Bentley 2005; Kuehner *et al.* 2011). The CTD consists of many tandem repeats of the heptapeptide YSPTSPS. Changes in the phosphorylation state of these residues at different stages of the transcription cycle affect the ability of Pol II to associate with other proteins, including various RNA processing factors (Buratowski 2005). These observations suggest a mechanism for recruitment of proteins required for termination or the loss of proteins required for processivity, as predicted by the antiterminator model and possibly also required as a component of the torpedo mechanism.

Much more mechanistic detail is known about transcription termination by other multisubunit RNAPs. For example, intrinsic termination by *Escherichia coli* RNAP requires a hairpin structure in the nascent RNA directly upstream of a stretch of uridines (von Hippel 1998; Peters *et al.* 2011). The hairpin promotes melting of the upstream edge of the weak DNA:RNA hybrid, facilitating dissociation of the remaining rU:dA base pairs and collapse of the transcription bubble (Gusarov and Nudler 1999; Komissarova *et al.* 2002). Termination by yeast Pol III appears to be even simpler, requiring a run of multiple adenosines in the template DNA but possibly independent of accessory proteins (Richard and Manley 2009).

Mutations that increase or decrease the response of *E. coli* RNAP to intrinsic terminators have been isolated in the *rpoB* and *rpoC* genes that encode the two largest subunits, β and β’, respectively (*e.g.*, Landick *et al.* 1990; Weilbaecher *et al.* 1994; reviewed in Trinh *et al.* 2006). In most cases, the affected residues were in regions of strong sequence homology to other prokaryotic and eukaryotic multisubunit RNAPs, suggesting that some general features of transcription termination are shared among these enzymes, even though the detailed mechanisms vary. Consistent with that idea, Shaaban *et al.* 1995 isolated termination-altering mutations in the second largest subunit of yeast RNA polymerase III (Pol III) by specifically targeting conserved areas shown to be important for *E. coli* RNAP termination.

In several studies investigators have demonstrated phenotypes consistent with termination defects for mutant alleles of *RPB1* and *RPB2*, the genes encoding the first and second largest subunits of yeast Pol II. (Cui and Denis 2003; Kaplan *et al.* 2005; Kaplan *et al.* 2012). In addition, mutations in the Rbp3 and Rpb11 subunits of yeast Pol II were obtained in an untargeted screen for increased terminator readthrough mutants (Steinmetz *et al.* 2006). However, a genetic screen specifically designed to isolate termination-altering mutations of Pol II has not yet been reported. To gain further insight into the role of Pol II in coupling polyadenylation to termination, we conducted such a screen and isolated mutants that showed an aberrant response to a well-characterized polyadenylation-dependent termination signal in *Saccharomyces cerevisiae*. We targeted the mutations to the upstream half of *RPB2* because the N-terminal portion of the Rbp2 subunit contains several regions of high sequence and structural similarity shown to be important for termination in other RNAPs, as well as fairly extensive regions that are conserved in but unique to eukaryotic Pol II enzymes (Sweetser *et al.* 1987). We describe the identification and initial characterization of 38 mutant *rpb2* alleles that confer either a decreased or increased response to one or more termination sites.

## Materials and Methods

### Yeast strains and plasmids

Standard techniques and media (Ausubel *et al.* 1988) were used for the yeast strains, which were derivatives of Research Genetics strain BY4742 (*MAT*α *his3*Δ*1 leu2*Δ*0 lys2*Δ*0 ura3*Δ*0*). DHY268 (BY4742 *trp1ΔFA rpb2Δ*::*HIS3* [pRP212]) was the background strain used for the initial screen and DHY349 (DHY268 *can1-100 cup1Δ*::*HYG*) for most of the experiments characterizing the mutant phenotypes.

pRP212 and pRP214 are *CEN*-based plasmids containing a wild-type copy of *RPB2* and a *URA3* or *LEU2* marker, respectively [gift from Richard Young, MIT (Scafe *et al.* 1990b)]. pRP214BX is a derivative of pRP214 that contains *BamH*I and *Xma*I restriction sites engineered into the *RPB2* open reading frame by site-directed mutagenesis. The silent mutations altered codons 207-208 (GGT/TCC changed to GGA/TCC) and 578-579 (ACA/AGG changed to ACC/CGG).

pL101Btrp, used to screen for termination-altering mutations, was derived from pL101 [a gift from Linda Hyman, Tulane University (Hyman *et al.* 1991)]. The *rp51-ADH2*p(A)-*lacZ* fusion reporter gene on pL101, a 2µ plasmid with a *URA3* marker gene, was amplified by polymerase chain reaction (PCR) and transferred to pRS414, a *CEN*-based plasmid with a *TRP1* marker. pD16trp, used as a positive control in the termination screen, was similarly modified from D16 (also from Linda Hyman) and is identical to pL101Btrp except that the reporter gene lacks the *ADH2* terminator (Hyman *et al.* 1991).

pGAC-CYC83Ftrp and pGAC-SNR13Ftrp were used to test the extent of readthrough of the *CYC1* and *SNR13* terminators. These *CEN*-based plasmids, in which the *CUP1* copper-resistance gene is used as a reporter for readthrough, were derived from pGAC-CYC83F and pGAC-SNR13F [provided by David Brow and Eric Steinmetz, University of Wisconsin, Madison (Steinmetz *et al.* 2001; Steinmetz and Brow 2003)] by replacing the *LEU2* marker gene with *TRP1*. These plasmids were introduced into DHY349-derived yeast strains bearing pRP214 (wild-type *RPB2*) or derivatives with *rpb2* mutant alleles, and the resulting strains were tested for growth on minimal media containing 150, 175, and 200 µM CuSO_4_ (for the *CYC1* terminator) or 350 and 400 µM CuSO_4_ (*SNR13* terminator).

For those and other growth tests, fivefold serial dilutions of log-phase cells were spotted onto minimal and/or rich medium and incubated at 30° unless otherwise indicated. The growth was scored relative to isogenic strains containing pRP214 with the *RPB2* gene. Mycophenolic acid (MPA) sensitivity was tested at 50 µM on minimal media.

### Random mutagenesis and screening strategy

Random mutations were introduced into the upstream half of *RPB2* using PCR with Taq polymerase and the DHO86 and Rpb2xbr primers (Supporting Information, Table S1). The purified PCR product (300 ng) and 100 ng of *BamH*I-*Xma*I−digested pRP214BX were cotransformed into DHY268 harboring pL101Btrp and plated onto glucose minimal media lacking Leucine and Tryptophan (SD-LEU-TRP). Individual *LEU2TRP1* transformants were patched to SD-LEU-TRP plates and cured of the wild-type copy of *RPB2* by negative selection on media containing 5-fluoroorotic acid (Boeke *et al.* 1984). Surviving cells were transferred to synthetic media with galactose to induce expression of the *lacZ* reporter gene.

*lacZ* expression was detected using an X-gal colony filter lift procedure. Patches were lifted from the plates with Whatman #5 filter paper (Sigma-Aldrich). The filters were submerged in liquid nitrogen for approximately 10 sec. Thawed filters were placed on a second filter soaked in 2 mL of X-gal Z-buffer (60 mM Na_2_HPO_4_, 40 mM NaH_2_PO_4_, 1 mM MgSO_4,_ 10 mM KCl, pH 7.0) with 38 mM β-mercaptoethanol and 400 µg/mL X-gal (Sigma-Aldrich). Color development was monitored until the control strain with the wild-type *RPB2* allele exhibited no further color change (generally several hours). The pRP214 derivatives that appeared to confer either increased or decreased terminator readthrough were isolated and reintroduced into yeast. Mutant alleles were sequenced if the change in *lacZ* expression was recapitulated in the reconstructed strains.

### cDNA analysis

Cells were grown in rich media to saturation, then diluted to an OD_600_ of 0.2 in 5 mL of YPGE (1% BactoYeast extract, 2% BactoPeptone, 2% glycerol, 2% ethanol) and grown to an OD_600_ of ~1.0. Total RNA was prepared by the hot acid phenol procedure (web.mit.edu/biomicro/forms/biofabmanual.pdf). Trace DNA contamination was eliminated using the Turbo DNA-free kit (Ambion) according to the manufacturer’s instructions. A 20-µL reaction containing 1 µg of RNA and 2 pmol random 9-mer primers was incubated at 70° for 5 min, then cooled on ice for 5 min. After the addition of deoxynucleoside triphosphates and dithiothreitol (final concentrations of 0.5 mM and 100 mM, respectively) and First-Strand Buffer (Invitrogen), incubation resumed at 42° for 2 min. Moloney murine leukemia virus reverse transcriptase (Invitrogen; 200 units) was added and incubation continued at 42° for 60 min, followed by heat inactivation for 15 min at 70°. The reaction was then incubated with 5 units of RNase H for 20 min at 37° and heat inactivated for 10 min at 65°.

Then, 2.0 µL of each cDNA reaction was used in two separate PCRs with a forward primer (BC117) and a reverse primer, either BC116 or BC130 (Table S1), at 1 pmol each in a 50-µL reaction containing 500 mM KCl; 100 mM Tris, pH 8.9; 1.0% Triton X-100; 2.5 mM MgCl_2_; 0.2 mM deoxynucleoside triphosphates; and 2.5 µL of Taq DNA polymerase. PCR products were resolved on a 1.2% agarose gel containing ethidium bromide.

In some experiments, specific primers BC118, complementary to the C-terminal portion of ADH2 open reading frame, and BC133, which anneals about 400 nt downstream of the *ADH2* poly(A) site, were used for cDNA synthesis instead of random primers (Table S1).

### Quantitative reverse transcription PCR (qRT-PCR)

RNA isolation and cDNA synthesis with random primers was as described previously. PCRs were performed in an ABI PRISM 7900HT in a total volume of 40 µL for 35 cycles, using the conditions described in (Rogatsky *et al.* 2003). The primers used are listed in Table S1. The generation of specific PCR products was verified by melting curve analysis and gel electrophoresis. Quantification of cDNA species was as described (Pfaffl 2001). *P* values comparing the results from each strain with the wild-type strain were calculated using the paired *t*-test (pairing wild-type and mutant reactions in the same 96-well plate). The cDNA levels were analyzed for each mutant strain in at least six independent experiments beginning with growth of cells and RNA isolation (File S1).

## Results

**Figure 1:**
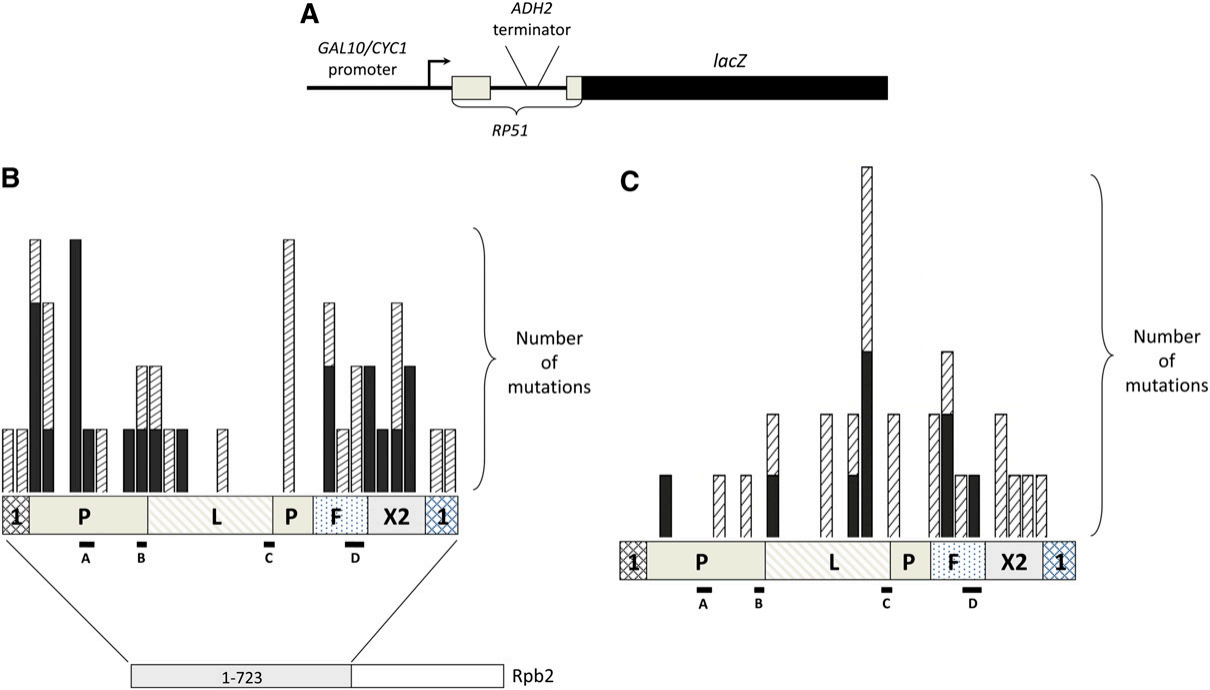
Termination screen reporter and distribution of amino acid substitutions. (A) Schematic of the termination reporter gene construct (not to scale) used in the screen (Hyman *et al.* 1991). (B) Distribution of amino acid substitutions associated with an increased readthrough (blue) phenotype. The N-terminal portion of Rpb2, in which mutations were introduced, is shown as a bar with different patterned intervals representing the defined structural regions (Cramer *et al.* 2001). These are: 1, external 1; P, protrusion; L, lobe; F, fork; and X2, external 2. The black lines below this bar indicate named regions of sequence homology among bacterial and eukaryotic RNAPs (Sweetser *et al.* 1987). The bar graph displays the number of mutations obtained in successive intervals of 20 amino acids. The solid bars represent amino acid substitutions that occurred either alone or in combination with another mutation in the same structural region. The striped portions denote substitutions that occurred in combination with another mutation in a different structural region. (C) Distribution of amino acid substitutions identified in *rpb2* alleles with a decreased readthrough (white) phenotype. The bar graph was constructed as in (B).

Our screen used a well-characterized reporter construct previously used to identify and characterize *cis*-acting sequences and *trans*-acting factors that contribute to polyadenylation and termination in yeast (Hyman *et al.* 1991; Magrath and Hyman 1999; Cui and Denis 2003; Bucheli and Buratowski 2005). This construct contains the yeast *ADH2* polyadenylation-dependent terminator in an intron upstream of the *E. coli lacZ* gene ORF (Figure 1A). Because the response to the poly(A) site is not 100% efficient and must occur before the intron is spliced, yeast colonies with wild-type Pol II make a small amount of β-galactosidase and consequently appear light blue when exposed to X-gal. The desired classes of Pol II mutations that increased or decreased the frequency of readthrough of the *ADH2* terminator would be expected among mutants with detectably darker blue or white colonies, respectively.

We generated random mutations by using PCR and replaced the wild-type copy of *RPB2* with the mutant alleles via plasmid shuffle in a yeast strain deleted for the chromosomal *RPB2* locus (Materials and Methods). Among approximately 2000 *rpb2* strains tested, we identified 100 strains with either increased or decreased levels of β-galactosidase relative to wild-type cells. To verify that the mutated *rpb2* alleles were responsible for the observed phenotypes, we isolated the plasmids from the candidate strains and reintroduced them into yeast. Upon retesting, 24 *rpb2* strains were confirmed to have an increased expression (blue) phenotype (Table 1), whereas 16 displayed decreased expression (Table 2).

**Table 1:** Properties and phenotypes of *rpb2* increased readthrough (blue) strains

	Growth*^a^*				Reporter Expression With Ferminator From:				Homology Region*^c^*	Sequence Identity*^d^*	Structural Homology*^e^*	Domain*^f^*
Mutation	30°	16°	38°	MPA	*ADH2**^b^*	None*^b^*	*CYC1**^a^*	*SNR13**^a^*				
E104G*^g^*	wt	wt	wt	<	>>>	ND	>	>	−	HDPS	S	Protrusion
D106G	wt	wt	wt	<	>>	ND	>	wt	−	HDPS	S	Protrusion
Y190C									−	P	S	Protrusion
G107D	wt	wt	wt	<	>	wt	wt	wt	−	HDP	S	Protrusion
***Q46R****^h^*	wt	ND	wt	wt	>>	ND	>>	>>	See Figure 5D	HDPS	TS	Protrusion
*Y57F*									−	−	TS	Protrusion
***Q46R***	wt	wt	wt	<	>>	ND	>>	>	See Figure 5D	HDPS	TS	Protrusion
*E80D*									−	−	U	Protrusion
E437K	wt	wt	wt	wt	>>	ND	>>	>	−	P	S	Protrusion
T500A									−	P	TS	Fork
M432T	wt	wt	wt	<<	>>	ND	>>	>	−	P	TS	Protrusion
T339A									−	−	−	Lobe
***V225M***	wt	wt	<<	wt	>>	ND	>	wt	−	HDPS	TS	Lobe
*D279G*									−	P	TS	Lobe
***G127D***	wt	wt	wt	<	>>	>	wt	wt	A	−	TS	Protrusion
***I205V***									B	HDPE	TS	Protrusion
***N206Y***	<<	<<	<<	<<	>>	wt	<<	<	B	HDPES	TS	Protrusion
*V225E*									−	HDPS	TS	Lobe
*R605G*										HDPS	S	External 2
S2P	wt	wt	wt	wt	>>	wt	wt	wt	−	P	U	External 1
D66N									−	P	TS	Protrusion
W31R	<	wt	—	wt	>>	wt	wt	>	−	HDPS	TS	External 1
L74W									−	−	U	Protrusion
***S45L***	wt	wt	wt	wt	>>>	ND	>>	>	See Figure 5D	E	TS	Protrusion
***K537R***									D	HDPS	S	Fork
K148R	wt	wt	wt	wt	>	ND	>>	wt	−	−	U	Protrusion
E438G									−	−	U	Protrusion
H518Q									D	PE	S	Fork
N484D	wt	wt	wt	wt	>>	wt	<	<<	−	HDP	TS	Fork
T491I	wt	wt	wt	wt	>	ND	>>	>	−	HDP	S	Fork
E245G	wt	<	wt	wt	>>	ND	>	<<	−	HDP	S	Lobe
T527A									D	HDPES	S	Fork
H648P									−	−	−	External 1
V547A	wt	wt	wt	wt	>>	ND	>	>>	−	HDPS	S	External 2
F557S	wt	wt	wt	wt	>>	wt	wt	wt	−	HDP	S	External 2
H572Y	wt	wt	wt	wt	>	ND	>	>	−	−	−	External 2
*F581L*									−	HDP	S	External 2
N583D	wt	wt	wt	wt	>>	wt	wt	>	−	HDPS	S	External 2
F421L										HDP	TS	Protrusion
N592S	wt	wt	wt	>	>	ND	—	wt	−	−	S	External 2
I669T									−	−	U	External 1
L603S	wt	wt	wt	wt	>	ND	<	<<<	−	HDP	S	External 2

ND, not determined; wt, wild type.

a Growth on plates of fivefold serial dilutions of log-phase cells was assessed relative to wild type after 2 (30° or MPA), 3 (16° or 38°), or 6 (*CUP1* reporter strains) d: <, minor but reproducible growth defect; << or <<<, moderate or severe growth defect, respectively; −, no discernible growth after 7 d; >, minor but reproducible growth improvement; >>, substantial growth improvement.

b The relative intensity of blue color in the filter lift assay was scored when the control strain was still white as follows: >, light blue; >>, medium blue; >>>, dark blue. For strains containing pD16Trp (control plasmid with no terminator), > indicates that blue color developed more rapidly than for the control strain.

c Refers to the regions of homology defined by Sweetser *et al.* 1987.

d Sequence identity to the corresponding residue is indicated for human (H), *Schizosaccharomyces pombe* (P), and *Drosophila melanogaster* (D) RNA polymerase II and for *Escherichia coli* (E), and *Sulfolobus solfataricus* (S) RNAPs.

e Structural homology was determined using jFATCAT (rigid) for two-way comparisons between *S. cerevisiae* Pol II (1Y1W) and RNAPs from *Thermus aquaticus* (T; 2GHO) and *Sulfolobus solfataricus* (S; 3HKZ). U, unstructured in crystal.

f Domain designations are from Cramer *et al.* (2001).

g Two independent isolates.

h Site-directed mutagenesis was used to create *rpb2* strains containing only the single mutation shown in italics. Bold-faced type indicates that the mutation had a phenotype with the *rp51-ADH2*p(A)-*lacZ* fusion reporter (Table 3). Mutations in italics but not bold-faced did not elicit either a blue or white phenotype when tested as single mutations.

**Table 2:** Properties and phenotypes of *rpb2* decreased readthrough (white) strains

	Growth*^a^*				Relative Reporter Expression With Terminator From:				Homology Region*^a^*	Sequence Identity*^a^*	Structural Homology*^a^*	Domain*^a^*
Mutation	30°	16°	38°	MPA	*ADH2**^b^*	None*^b^*	*CYC1**^a^*	*SNR13**^a^*				
A75T	wt	wt	wt	wt	<	wt	wt	wt	−	HDP	U	Protrusion
Y149D	wt	wt	wt	<	<<	ND	>	<	−	−	U	Protrusion
D568G									−	P	S	External 2
K191M	wt	wt	<	<<<	<<	wt	<	<	−	−	S	Protrusion
***I343T****^c^*									−	−	−	Lobe
N221K	wt	wt	wt	wt	<	wt	wt	wt	−	HDPS	TS	Lobe
E371G									−	HDP	TS	Lobe
S235P	<<<	−	−	<<<	<	ND	<<<	−	−	−	TS	Lobe
S480P									−	HDPSE	TS	Fork
N610S										−	S	External 2
D304V	wt	<	<<	<<<	<	<	<	<	−	HDPS	S	Lobe
***E368K***									−	P	TS	Lobe
L566P										HDP	S	External 2
V305I	wt	ND	wt	<	<	ND	<<	−	−	−	S	Lobe
F376S									−	P	TS	Lobe
***F581S***										HDP	S	External 2
D407G	wt	wt	wt	wt	<	ND	wt	wt	−	HDP	TS	Protrusion
E468G									−	−	−	Fork
K625N									−	−	S	External 2
I343T	wt	wt	wt	<<<	<	wt	<<	<<<	−	−	−	Lobe
L361P	<	wt	<<<	<<<	<	wt	<	<<<	−	HDPS	S	Lobe
***E368G***	wt	<	wt	<<<	<<	<	<<<	<<	−	P	S	Lobe
I502T									−	HDP	TS	Fork
E368K	wt	<<	wt	<<<	<<	wt	<<<	<<	−	P	S	Lobe
K418M	<	<<	<	wt	<	wt	<<<	−	−	−	TS	Protrusion
S489P									−	−	TS	Fork
Q481R*^d^*	wt	wt	<	<<	<<	<	<<<	<<	−	HDPES	TS	Fork
K537E	wt	wt	<<	wt	<<	ND	<<<	−	D	HDPS	TS	Fork

ND, not determined; wt, wild type.

a Same as in Table 1.

b The relative intensity of blue color was scored as follows: <, light blue relative to the blue color observed for the control; <<, remaining white at the end of the assay.

c Strains with the single mutations in bold italics were shown to have phenotypes with the *rp51-ADH2*p(A)-*lacZ* reporter (Table 2, 3, or 4).

d Two independent isolates.

All but two of the *rpb2* blue alleles were unique; E104G was obtained twice (Table 1). One amino acid substitution (Q46R) occurred in two alleles with different second mutations. Construction and analysis of the corresponding single mutants confirmed that the Q46R mutation caused the blue phenotype in both of the isolated alleles (Table 3). One position (V225) was mutated to two different amino acids, but only one of these substitutions conferred a blue phenotype as a single mutation (Table 3).

**Table 3:** Phenotypes of site-directed *rpb2* mutants

	Growth*^a^*				Reporter Expression With Terminator From:		
Mutation	30°	16°	38°	MPA	*ADH2*	*CYC1**^a^*	*SNR13**^a^*
S45L	wt	wt	wt	ND	Blue	ND	ND
Q46R	wt	ND	ND	ND	Blue	>>	>>
Q47R	wt	wt	wt	wt	Blue	>>	>>
Y57F	wt	wt	wt	wt	wt	wt	wt
E80D	wt	>	wt	wt	wt	wt	wt
R120C	<	<<	−	−	Blue	<<	<<
G127D	wt	wt	wt	ND	Blue	ND	ND
I205V	wt	wt	wt	ND	Blue	ND	ND
N206Y	wt	wt	wt	wt	Blue	wt	wt
V225E	wt	wt	wt	wt	wt	wt	wt
V225M	wt	wt	wt	wt	Blue	>	wt
D279G	wt	wt	wt	wt	wt	wt	wt
F581L	wt	wt	wt	wt	wt	wt	wt
F581S	wt	wt	wt	wt	Blue	wt	>>
R605G	wt	ND	ND	ND	wt	ND	ND

ND, not determined; wt, wild type.

a As described for Table 1.

There were 15 unique white mutants; two alleles were the same (Q481R; Table 2). Two substitutions (I343T and E368K) were isolated twice, in each case both as a single mutation and also in combination with additional mutations. We also isolated a different substitution at position 368 (E368G).

Figure 1, B and C shows the locations of the amino acid substitutions with respect to the Rpb2 structural domains defined by Cramer *et al.* (2001) from the crystal structure of yeast Pol II. The great majority of the amino acid substitutions found in the blue mutants occurred in three domains: the protrusion, external 2, and the fork (Figure 1B). Indeed, every Rpb2 variant except one was affected in one or more of those domains, which together comprise only about 55% of the mutagenized area (Figure 1B). Only four mutations were isolated in the lobe; of those, only one (V225M) was shown to be responsible for the blue phenotype (Tables 1 and 3).

In contrast, more than half of the white mutants contained at least one amino acid substitution in the lobe (Figure 1C). Relatively few white mutations occurred in either the external 2 or protrusion domains, and all but two of those were accompanied by mutations in the lobe and/or fork domains. Mutations in the fork were associated with both phenotypes. Indeed, mutations at K537 were found in both a blue (K537R) and a white (K537E) allele (Tables 1 and 3). We also found mutations affecting F581 in the external 2 domain in both blue and white alleles. Both F581 mutations were isolated in combination, so we constructed *rpb2* alleles containing the single mutations (Table 3). The mutation in the white allele, F581S, conferred a blue phenotype, demonstrating that at least one of the other two mutations in that allele was responsible for the white phenotype. The conservative mutation in the blue allele, F581L, conferred a wild-type phenotype with the *lacZ* reporter, implicating the other external 2 mutation in that allele (H572Y) as the source of the blue phenotype.

### Tests for fitness and altered function at other terminators

Only five mutants had discernible growth defects at 30°, and white mutants were much more likely than blue mutants to be heat- or cold-sensitive (Tables 1 and 2). Most of the white mutants also were sensitive to MPA, a drug that inhibits *de novo* synthesis of GMP. MPA sensitivity in yeast often is associated with mutations that alter Pol II elongation activity and/or transcriptional start site selection (Powell and Reines 1996, Shaw and Reines 2000, Shaw *et al.* 2001, Desmoucelles *et al.* 2002, Kaplan *et al.* 2012).

**Figure 2:**
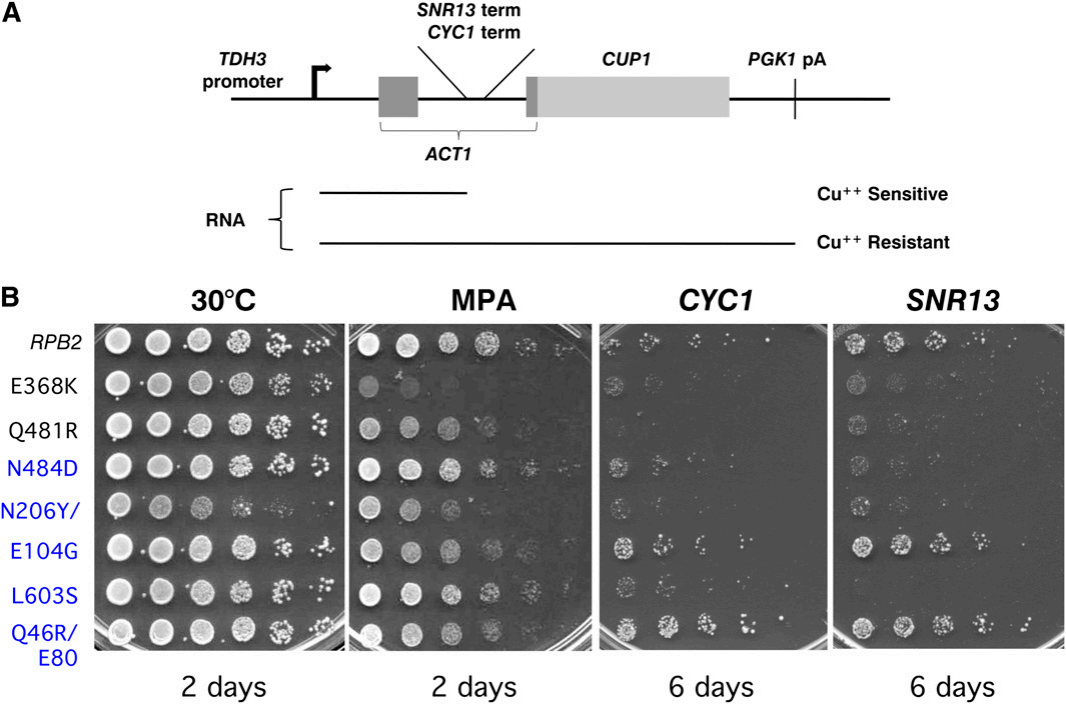
Termination defects at the *CYC1* and *SNR13* terminators. (A) Schematic representation of the *CUP1* reporter constructs used to investigate the generality of the termination defects caused by *rpb2* mutations (Steinmetz *et al.* 2001; Steinmetz and Brow 2003). (B) Representative tests of the relative MPA sensitivity and *CUP1* reporter expression of *rpb2* yeast strains. The number of days of growth is shown. The CuSO_4_ concentration was 200 µM and 400 µM for the constructs containing the *CYC1* or *SNR13* terminator, respectively. The amino acid changes in the Rpb2 mutants are shown except for the triple mutant N206Y/V225E/R605G (N206Y/ in B). Blue lettering indicates mutations that resulted in a blue phenotype with the *rp51-ADH2*p(A)-*lacZ reporter*; black lettering indicates white mutants.

We tested the propensity of the Pol II variants to read through two additional terminators *in vivo* using well-characterized reporter constructs containing the poly(A)-dependent *CYC1* termination sequence and the poly(A)-independent *SNR13* termination sequence (Steinmetz *et al.* 2001; Steinmetz and Brow 2003). The reporter gene is an *ACT1:CUP1* fusion transcribed from a strong constitutive promoter (Figure 2A). Termination sequences embedded in the *ACT1* intron normally prevent the reporter gene from complementing a chromosomal *cup1* deletion, resulting in copper sensitivity (Steinmetz *et al.* 2001). We tested the *rpb2* strains on a range of copper concentrations so that we would be able to identify both increased and decreased sensitivity to copper (Materials and Methods).

We found that more than two-thirds of the blue *rpb2* strains grew better on copper than the control strain when the intron contained either the *CYC1* or the *SNR13* terminator, consistent with enhanced readthrough of those sites (Figure 2B and Table 1). In general, blue strains that were less copper sensitive with one terminator showed a similar response to the other. As expected, most of the white strains grew poorly on copper compared to the wild-type strain, consistent with an enhanced response to both of the terminators (Figure 2B and Table 2).

A few of the blue and white strains appeared wild-type in response to both termination sites, suggesting that their defects were specific to—or more sensitive to—a specific property of the *ADH2* site. This outcome would not be surprising, as varying responses to different termination sites have also been observed for termination mutants of other RNAPs (*e.g.*, Landick *et al.* 1990, Tavormina *et al.* 1996a). However, we also considered the possibility that the phenotypes of some mutant strains were not directly or solely due to aberrant behavior at the *ADH2* terminator but might instead reflect a change in the level of induction of the *GAL10/CYC1* promoter, the speed or efficiency of RNA splicing, or the ability of the Pol II to elongate through the *lacZ* gene.

To test whether the *ADH2* terminator was required for the altered *lacZ* expression, we repeated the β-galactosidase tests using a control plasmid (pD16trp) that was identical to the reporter plasmid used in the screen, except that the intron did not contain a terminator. We tested most of the white mutants and some of the blue mutants, including those that had a wild-type response to the *CYC1* and *SNR13* terminators. All of the strains turned blue in this assay, and all but four did so at the same time as the strain containing wild-type *RPB2* (Tables 1 and 2 and data not shown). Three white *rpb2* strains required about twice as long as the wild-type to turn dark blue with the reporter gene lacking the poly(A) site. However, these same strains had remained totally white after much longer assay times with the original reporter construct, showing that the poly(A) site was required for the white phenotype.

These results support the conclusion that the blue/white phenotypes reflected an aberrant response to the *ADH2* terminator for some or all of the *rpb2* alleles. However, since the intronic location of the poly(A) site in the reporter plasmid dictated that cleavage and splicing were mutually exclusive outcomes, a change in the rate of splicing remained a possible explanation, especially for the white mutants. A reduced rate of splicing could increase the time during which the poly(A) site was available for recognition and cleavage, potentially resulting in fewer transcripts that escaped premature polyadenylation (white phenotype).

### Blue mutants show reduced cleavage efficiency at the chromosomal *ADH2* poly(A) site

We considered decreased efficiency of RNA cleavage at the *ADH2* poly(A) site to be the most likely cause of the blue phenotype. Any scenario that allowed cleavage at the poly(A) site would prevent translation of the resulting, uncapped RNA. Therefore, other Pol II behaviors, such as enhanced elongation through *lacZ* sequences, would increase *lacZ* expression only from those transcripts that were spliced before cleavage occurred. A change in the rate of RNA splicing was also possible; increased *lacZ* expression would presumably require a faster rate of splicing to decrease the time available for poly(A) site use.

**Figure 3:**
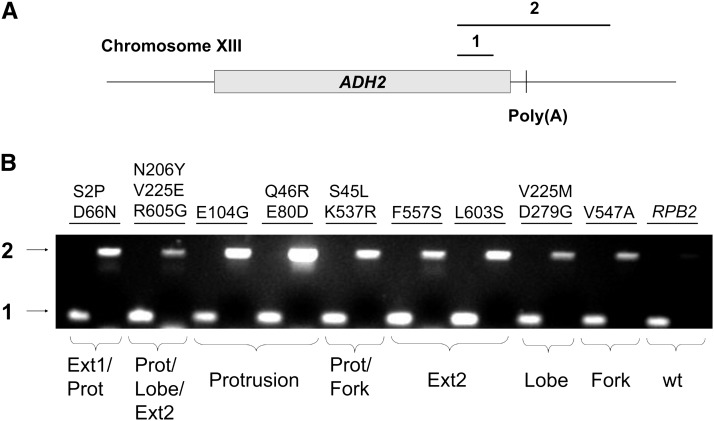
cDNA analysis of readthrough at the *ADH2* locus. (A) A schematic view of the *ADH2* locus and the expected products of the PCR reactions are shown. Total RNA isolated from strains containing the indicated *rpb2* alleles was used to synthesize cDNAs from random primers. The cDNAs were then amplified in separate PCR reactions using primers corresponding to PCR products 1 and 2. (B) The products of PCR amplication of the cDNAs were electrophoresed on an agarose gel. The domains that were affected by the mutations are indicated below the gel.

The intronic location of the poly(A) site in the reporter construct precluded measurement of steady state levels of uncleaved transcripts. To directly examine the extent of RNA cleavage in response to the *ADH2* poly(A) site, we instead monitored RNA synthesized from the chromosomal *ADH2* locus (Figure 3A). Total RNA was isolated from the wild-type strain and nine representative blue strains grown in media that induced the *ADH2* promoter (see *Materials and Methods*). cDNAs synthesized using random primers were amplified in two separate PCRs. The upstream PCR primer for both reactions annealed within the ORF. The downstream primer in the PCR1 reaction hybridized upstream of the stop codon, so all of the RNA species of interest would contribute to a product, regardless of whether the RNAs were properly cleaved and/or terminated. The downstream primer in the PCR2 reaction annealed more than 200 nt 3′ of the most distal sites at which polyadenylation-associated cleavage has been shown to occur (Hyman *et al.* 1991). Only cDNAs corresponding to uncleaved RNAs from elongation complexes that had escaped the normal termination interval would be amplified in that reaction.

We tested cell lines harboring *rpb2* blue alleles encoding substitutions in each of the different structural regions. Most strains showed a significant increase in the ratio of PCR2 to PCR1 relative to wild-type cells (Figure 3B). That result both confirmed an increased steady-state level of uncleaved transcripts and also demonstrated that the aberrant behavior did not depend on features of the reporter construct (*e.g.*, the intron) that were not shared by the chromosomal *ADH2* gene. The triple mutant N206Y/V225E/R605G was a possible exception, as the PCR2 product was not as enriched relative to PCR1 as was seen for the other mutant stains. That strain also differs from the other blue mutant strains in having a pronounced growth defect (Table 1 and Figure 2).

We repeated these experiments for several mutants using cDNAs synthesized from specific, rather than random, primers to eliminate the possibility that the RNA spanning the poly(A) site arose from an antisense transcript (see *Materials and Methods*). The method of cDNA priming did not change the qualitative outcome or interpretation of the PCR reactions (Figure S1).

### Correlation between poly(A) site cleavage and termination

The design of primer sets used in the experiment of Figure 3 precluded detection of RNAs that had been cleaved but not terminated or terminated without being cleaved. Therefore, that experiment did not reveal whether any of the mutations had altered the normal coupling between the polyadenylation and termination. We used qRT-PCR to address this issue by measuring separately the amount of uncleaved and readthrough transcripts from the *ADH2* gene.

**Figure 4:**
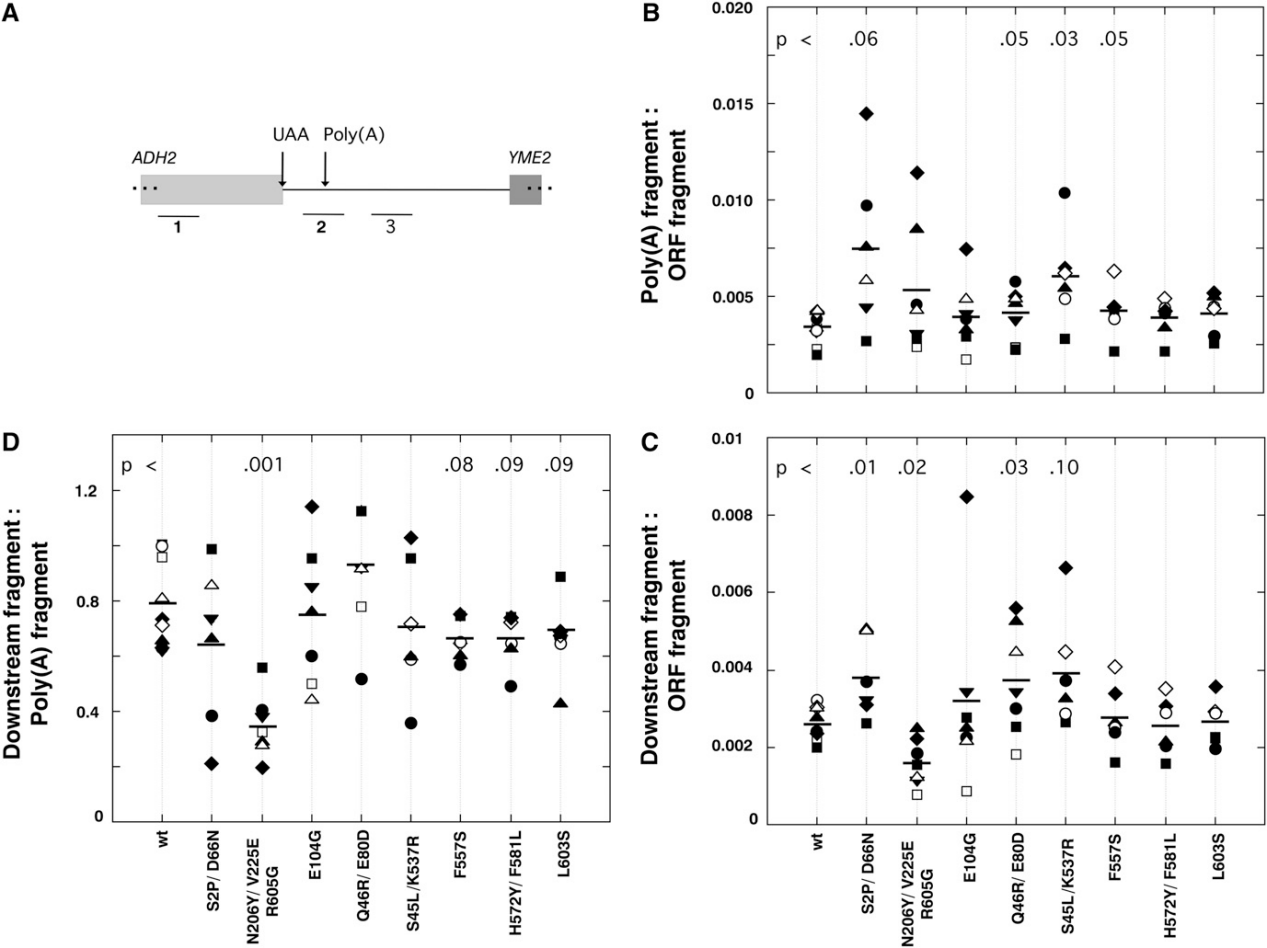
qRT-PCR to assess cleavage and readthrough of the *ADH2* terminator. (A) *ADH2* cDNAs synthesized using random primers were analyzed with three sets of primers to amplify the 120-bp regions shown below the gene diagram. (B) Results of qRT-PCR are presented as a ratio of the amount of poly(A) site cDNA to the ORF cDNA product. Various symbols represent different RNA preparations; the same symbol is used for qRT-PCRs performed in the same 96-well plate. Horizontal bars indicate averages of the 6 or more experiments for each strain. P values ≤ 0.1 are indicated. (C) Same as in B, except that downstream cDNA is compared with the ORF cDNA. (D) Same as in B, except that the downstream cDNA is compared to the poly(A) site cDNA in each experiment.

We used the primer sets shown in Figure 4A to monitor three cDNA regions: the ORF, the poly(A) site, and a sequence more than 300 bp downstream of the poly(A) site. In each experiment, we calculated the ratio of poly(A) site or downstream PCR product to the ORF (total RNA) product (Figure 4, B and C). Measurements of the relative PCR efficiencies indicated that all three primer sets yielded close to the same amount of PCR product (±10%) when used to amplify DNA spanning the entire region (data not shown). Therefore, the numbers on the *y*-axis are close to true ratios. There were no systematic differences among the wild-type and mutant strains in the amount of PCR fragment corresponding to the ORF, indicating that none of these mutations affected transcription initiation at the *ADH2* promoter (data not shown).

The steady-state accumulation of uncleaved RNAs is shown in Figure 4B. For the wild-type strain, approximately 0.3% of the transcripts containing the *ADH2* ORF were uncleaved at the poly(A) site. The average amount of poly(A) fragment was slightly increased over the wild type for all of the mutants, although in most cases the difference was just outside what is normally considered statistically significant (*P* < 0.05). The highest ratio—just greater than twofold when the average value was compared with wild-type—was observed for the S2P/D66N mutant. The modest increases in uncleaved poly(A) site RNA are consistent with expectation, because only one blue mutant (N206Y/V225E/R605G) had a severe fitness defect at 30° (Table 1). We noted that the distribution of values among different experiments was much greater for some of the mutants than for wild type, possibly because some of the Pol II mutations had stochastic effects (*e.g.*, by influencing the steady state levels of polyadenylation machinery components).

The relative steady state levels of the downstream fragment are shown in Figure 4C. The results of this comparison were more varied, with several mutants appearing similar to wild-type, several with a higher accumulation of this fragment (*e.g.*, S2P/D66N), and one (N206Y/V225E/R605G) with a significantly reduced accumulation. The result suggested that one or more of the mutations had affected the correlation between uncleaved RNA and readthrough into downstream sequences. To make that direct comparison, we plotted the ratio of downstream fragment to uncleaved poly(A) site fragment (Figure 4D). For the wild-type strain, that value was about 0.8, consistent with models suggesting that cleavage is required for termination. For half of the tested mutants, this ratio was statistically the same as for the wild-type strain. For one mutant strain, N206Y/V225E/R605G, we observed a highly significant decrease in the ratio. This result suggested that poly(A) site usage and downstream termination were at least partially uncoupled in this strain, in that the reduced efficiency of poly(A) site usage did not result in increased accumulation of downstream RNA. It is possible that the reduced fitness of this strain is related to this unique behavior.

A smaller—but possibly significant—decrease in the ratio of downstream:uncleaved RNA was noted for three additional mutant strains (Figure 4D). Although the *P* values were greater for these strains than for the triple mutant strain (0.08 and 0.09 compared with 0.001), they are strikingly different from the *P* values determined for the rest of the tested mutants (>0.8). Interestingly, all of these mutants, including the triple mutant, have substitutions in the external 2 domain.

### Mutations in phylogenetically conserved residues

**Figure 5:**
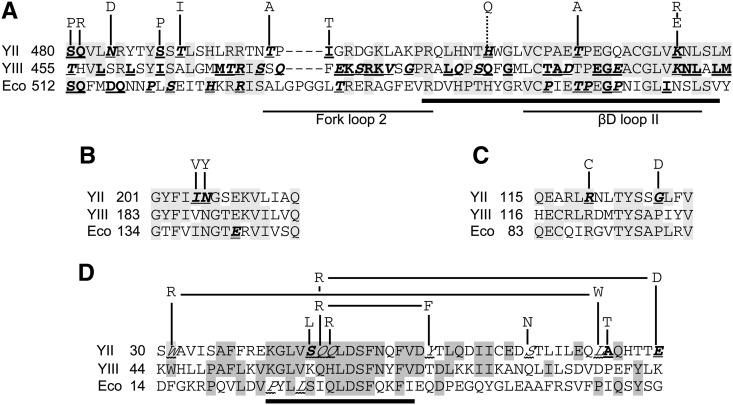
Amino acid substitutions in phylogenetically conserved regions. (A) Amino acid sequences are shown for a portion of the fork domain of *S. cerevisiae* Rpb2 (YII) and the corresponding sequences of the Ret1 and β subunits from *S. cerevisiae* RNA polymerase III (YIII) and *E. coli* (Eco), respectively. Shading indicates amino acids that are identical in at least two of the three aligned sequences. The thick line below the sequences indicates residues within this interval that are part of homology block D (Sweetser *et al.* 1987). *rpb2* substitutions identified in this study are shown above the alignment; the dotted line indicates a mutation that has been tested only in combination with an additional substitution. Underlining indicates positions at which termination-altering mutations were isolated for Rpb2 (this study), Ret1 (Shaaban *et al.* 1995), and the β subunit (Jin *et al.* 1988; Landick *et al.* 1990; Tavormina *et al.* 1996a). Italics with wavy underlining indicate residues mutated in increased readthrough variants, whereas bold-faced type with straight underlining indicate decreased readthrough variants. One fork mutation, affecting E468 in fork loop 1 (Table 2), is not shown. (B) Mutations affecting homology region B. The notation is as in (A). The double-underlined residue E142 in the *E. coli* sequence was identified as a second site suppressor of an increased termination mutant (Tavormina *et al.* 1996b). (C) Mutations affecting homology region A. The R120C mutation was originally isolated as the heat- and cold-sensitive *rpb2-7* allele (Scafe *et al.* 1990a). (D) Amino acid sequences are shown for the N-terminal regions with the same notation as in (A). Rpb2 single and double substitutions isolated within this interval are shown above the sequence. The two underlined residues in the *E. coli* sequence were mutated in a recessive lethal allele of *E. coli rpoB* associated with enhanced readthrough of some terminators (Tavormina *et al.* 1996a). The thick line below the sequences shows the region of homology defined by Lane and Darst (2010).

**Figure 6:**
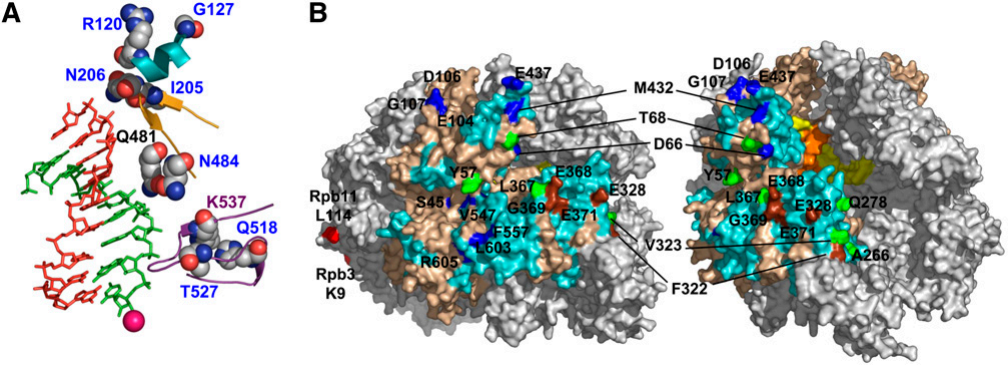
Location of mutated residues in the Pol II structure. (A) Mutated residues located close to the DNA:RNA hybrid in the crystal structure of a Pol II elongation complex are shown (carbon, gray; nitrogen, blue; oxygen, red). Homology regions A, B, and D are depicted as teal, orange, and violet ribbons. RNA and DNA are shown in green and red, respectively. The active site Mg^++^ is depicted as a magenta sphere. All of the mutated residues were associated with blue alleles, except for Q481 (white) and K537 (both blue and white). This figure was created from pdb file 1I6H using PyMOL (DeLano Scientific). (B) The residues of Rbp2 are shown in tan, except for the residues that closely approached TFIIF in the PIC, as determined by Hahn and colleagues (Chen *et al.* 2007, Eichner *et al.* 2010), which are colored cyan. The Rpb2 positions indicated in green were found to crosslink to TFIIF (Chen *et al.* 2007). Surface residues mutated in Rpb2 variants that increased or decreased readthrough of the *ADH2* terminator are shown in blue and brown, respectively. Surface residues in Rpb3 and Rpb11 that were identified in a separate study of Pol II termination mutants (Steinmetz *et al.* 2006) are red. The rest of the Pol II subunits are gray. The view on the right is oriented so that part of the DNA:RNA hybrid in the active site is visible; the RNA is orange and DNA is yellow.

The mutagenized portion of *RPB2* contains four regions (homology blocks A-D) in which the sequences are highly conserved in all multisubunit RNAPs (Figure 5). Residues within these conserved regions closely approach nucleic acids in the elongation complex (Figure 6A). We isolated a number of mutations that altered residues in and around all of the homology blocks except C (Figure 5). Figure 5A shows the mutations located in the fork domain, in and immediately adjacent to homology block D. Many mutations that alter the termination behavior of *E. coli* RNAP and yeast RNA polymerase III have previously been isolated in the same region (Figure 5A).

We also isolated mutations in two residues that are among the most highly conserved positions in homology region B (Figure 5B and 6A). Both mutations, I205V and N206Y, were recovered in combination with other mutations. To determine whether the B region mutations were responsible for the termination phenotype, we used site-directed mutagenesis to create *rpb2* alleles containing the individual changes. The N206Y mutant had a blue phenotype, like the triply mutant strain, whereas both the *rpb2*-V225E and *rpb2*-R605G strains expressed the *lacZ* reporter at wild-type levels (Table 3). The finding that the V225E mutation did not confer a blue phenotype was interesting, since another mutation at the same position, V225M, did (Table 3). Similarly, the R605G mutation did not, by itself, confer a phenotype with the *lacZ* reporter, although L603S did (Table 1). One or both of these mutations had to have contributed to the growth defect of the triple mutant, since that property was not shared by any of the singly mutant strains (Table 3). It is likely, therefore, that one or both of these mutations also enhanced the excess readthrough defect caused by the N206Y mutation.

The I205V mutation was isolated in combination with a second mutation (G127D) that altered a highly conserved residue in homology region A (Figure 5C). Construction and testing of the two single mutants showed that both alleles caused a blue phenotype (Table 3). Besides G127D, only one other yeast *rpb2* region A mutation has been reported, R120C, which was isolated in the Young laboratory in a screen for conditional mutants (Scafe *et al.* 1990a). Previous studies of that allele (*rpb2-7)* have been somewhat equivocal but have suggested weak changes in the extent of readthrough of poly(A) sites (Cui and Denis 2003; Kaplan *et al.* 2005). In our assay strain, R120C conferred a blue phenotype (Table 3).

Finally, several of the blue strains had mutations affecting residues within a region of highly conserved sequence that was originally noted by James *et al.* 1991 and more recently identified in a comparison of more than 1000 bacterial, archaeal, and eukaryotic RNAP subunits (Figure 5D) (Lane and Darst 2010). Both S45L and Q46R were isolated in combination with other mutations. We constructed the single mutants and also an additional *rpb2* allele containing the same substitution at the neighboring position (Q47R). Each of these three mutations caused a blue phenotype (Table 3).

### Mutations in the TFIIF binding surface of the Rpb2 lobe cause a white phenotype

Most of the *rpb2* mutations altered residues clustered on the surface of Pol II in patches that likely coincide with binding sites for proteins involved in RNA processing and/or termination (Figure 6B). We have not yet identified the proteins that interact with the presumptive binding sites identified by mutations in the protrusion and external 2 domains of Rpb2. However, we observed that many of the mutations isolated in the lobe domain corresponded to or were near residues reported to interact with TFIIF, an essential transcription factor with proposed functions in both initiation and elongation (reviewed in Shilatifard *et al.* 2003; Chen *et al.* 2010).

The Hahn laboratory has identified positions in Rpb2 that crosslink to TFIIF when substituted with the synthetic, cross-linking residue BPA (Chen *et al.* 2007). Based on that information, they mutated specific residues and assayed the ability of the mutated Pol II to interact with TFIIF when assayed by coimmunoprecipitation (Chen *et al.* 2007). Two of the Rpb2 residues shown in that study to interact with TFIIF, E368 and E371, were mutated in our screen in three alleles that conferred a white phenotype (Table 2). We also isolated mutations that altered residues that were sites of cross-linking to TFIIF (Y57, L74) or next to sites of cross-linking in the primary sequence (A75, E468).

To determine whether alteration of the wild-type interaction between TFIIF and Pol II would cause a phenotype in our termination screen, we tested *rpb2* strains containing the mutations shown by Chen *et al.* to affect TFIIF binding *in vitro* (Table 4). All of those mutations shift transcription start sites upstream of where they occur in the wild-type strain (Chen *et al.* 2007), a property also reported for yeast with TFIIF subunit mutations (Ghazy *et al.* 2004; Freire-Picos *et al.* 2005; Eichner *et al.* 2010). Consequently, we tested two other previously reported mutations in the same area of the Rpb2 lobe: G369S, which causes a similar start site shift (Chen and Hampsey 2004), and G369D, which was isolated in a screen for *rpb2* strains with altered transcription initiation start sites (Hekmatpanah and Young 1991). A second mutation isolated in that same screen, E368K, was isolated twice in our study, as well, once in combination and once as a single mutation (Table 2).

**Table 4:** Phenotypes of *rpb2* mutations affecting residues important for TFIIF binding

	Growth*^a^*				Reporter Expression:			
Mutation	30°	16°	38°	MPA	*ADH2*	*CYC1**^a^*	*SNR13**^a^*	Reference
F322R	wt	ND	ND	ND	White	ND	ND	Chen *et al.* 2007
E328R	wt	ND	ND	ND	White	ND	ND	Chen *et al.* 2007
E368K	wt	<<	wt	<<<	White	<<<	<<	this study (Table 2) Hekmatpanah and Young 1991 (*rpb2-503*)*^b^*
E368G	wt	wt	wt	<	White	ND	ND	this study*^c^*
E368R	wt	ND	ND	ND	White	ND	ND	Chen *et al.* 2007
G369D	wt	<<	wt	<	White	<<	<<	Hekmatpanah and Young 1991 (*rpb2-504*; *rpb2-505)*
G369R	wt	<<	wt	<<	White	<<	—	Chen *et al.* 2007
G369S	wt	<<	wt	<<	White	<<	<<	Chen and Hampsey 2004 (*rpb2-101*)
E371R	wt	ND	ND	ND	White	ND	ND	Chen *et al.* 2007

ND, not determined; wt, wild type.

a As described for Table 1.

b Allele names associated with the mutations are provided following references to the articles in which they were reported.

c E368G was isolated with a second mutation (Table 2) and was separated from that mutation by site-directed mutagenesis. The resulting singly mutant strain was tested for phenotypes.

All of the *rpb2* mutants chosen for testing because of a demonstrated or hypothesized effect on TFIIF interactions had a white phenotype with the *lacZ* reporter (Table 4). A subset of mutations subjected to additional tests shared other common phenotypes, including MPA sensitivity and severe growth defects on copper in assays with the *CUP1* reporter constructs containing the *CYC1* and *SNR13* terminators. These properties were also shared by other white strains with mutations in nearby residues of the lobe domain (*e.g.*, I343T, L361P, and F376S; Table 2). These results suggest that mutations within this cluster of lobe residues confer a similar defect responsible for the decreased readthrough phenotypes. Based on published analyses of some of the mutants, that defect might involve an altered interaction with TFIIF.

## Discussion

The screen reported here proved a successful strategy for isolating *rpb2* alleles that alter the normal response of yeast Pol II to the poly(A)-dependent *ADH2* terminator, resulting in a collection of strains with increased or decreased readthrough phenotypes. Most of the mutant strains appeared to have mild but general termination defects, in that they also displayed similarly aberrant responses to another poly(A)-dependent site (*CYC1* terminator), a poly(A)-independent site (*SNR13* terminator), or both.

Analysis of the excess readthrough (blue) mutants verified that the screen had identified Pol II residues that contributed to the efficiency of cleavage at the chromosomal *ADH2* poly(A) site (Figure 3). Some of the mutations also may have interfered with the normal coupling of cleavage and subsequent termination (Figure 4). The fact that the mutations caused enhanced expression of the *lacZ* reporter is evidence that they did not also confer elongation or splicing defects, unless those activities were inappropriately enhanced.

In contrast, the decreased readthrough (white) strains could have defects in other transcription-related processes, including splicing and elongation. We were particularly aware of the latter possibility. Despite the wide-spread use of *lacZ* as a reporter in yeast, there are potential concerns when using a bacterial gene, which might contain cryptic processing sites (Cui and Denis 2003). In addition, because of the length of the ORF (> 3000 nt), *lacZ* expression might be especially sensitive to minor changes in Pol II elongation competency. However, we found that all but two of the mutants were indistinguishable from the wild-type strain in the level of expression of the *lacZ* gene when the reporter construct lacked the poly(A) site (Table 2). Furthermore, all but three of the white strains also showed deficiencies with a different reporter gene, the *ACT1:CUP1* constructs containing different yeast terminators (Figure 2 and Table 2). In contrast to *lacZ*, *CUP1* is a very short yeast gene with an ORF < 200 nt. Together these results strongly support the conclusion that both the blue and white mutants showed altered termination behaviors. Possible alterations to other properties, such as splicing efficiency and transcription elongation, if they occurred, were not sufficient to elicit the observed phenotypes. However, such altered behaviors might have contributed to the aberrant response to the poly(A) site.

A similar, although untargeted, screen for mutations causing excessive readthrough of Pol II terminators previously identified several mutations in different Pol II subunits, Rpb3 and Rpb11, the yeast homologs of the two alpha subunits of bacterial RNAP. In those experiments, Brow and colleagues used their *ACT1:CUP1* reporter construct containing the *SNR13* terminator (Figure 2A) to isolate spontaneous mutations in protein-encoding genes that conferred copper resistance (Steinmetz *et al.* 2006). The mutations altered surface exposed residues on the same side of the polymerase structure as the nearest amino acids mutated in our study but separated from them by more than 60 Å (Figure 6B). It is likely, therefore, that the two studies have located binding sites for different elongation, termination, or processing factors.

### Comparison with mutations affecting termination in other systems

In a previous screen for termination-altering mutations affecting the *E. coli* RNAP β subunit, the majority of mutations clustered in four regions, corresponding to parts of the lobe, the fork, and the hybrid-binding domain (Landick *et al.* 1990). Mutagenesis targeted to the corresponding regions of the yeast Pol III Ret1 subunit also resulted in termination phenotypes (Shaaban *et al.* 1995). The portion of Rpb2 that was mutagenized in our study contained two of these regions, the lobe and the fork. We isolated mutations in both of these locations (Figure 1, B and C). Most striking, all but two of the *rpb2* alleles that decreased readthrough had mutations affecting the lobe or the fork (Table 2). We also observed fork mutations, but very few lobe mutations, among the increased readthrough mutants (Figure 1B and Table 1). More than half of the fork mutations affected positions that were also mutated in termination-altering variants of either the *E. coli* β or yeast Ret1 subunit (Figure 5A). The high degree of sequence and structural conservation of these active site residues suggest that they have a common function in all RNAPs and may contribute to the termination defects in similar ways, despite the different mechanisms of termination used in the three systems.

The fork is composed of a series of loops that closely approach the DNA:RNA hybrid in the active site: fork loop 1, which is not present in bacterial RNAPs; fork loop 2, which is conserved among all multisubunit polymerases; and βD loop II, which was defined for the bacterial enzymes and includes part of the conserved D region (Korzheva *et al.* 2000; Gnatt *et al.* 2001; Trinh *et al.* 2006). We isolated mutations in each of these loops (Figure 5A). The mobility of the fork loops and their locations within the active site have suggested various functions during elongation, including maintaining and stabilizing the transcription bubble and promoting substrate binding, catalysis, and translocation (Trinh *et al.* 2006; Vassylyev *et al.* 2007; Kireeva *et al.* 2011).

Biochemical analyses of bacterial and Pol III systems *in vitro* have shown that fork domain substitutions can affect both pausing and the overall rate of elongation (Fisher and Yanofsky 1983; Landick *et al.* 1990; Shaaban *et al.* 1996; Tavormina *et al.* 1996b). Abnormally long pauses and slow polymerization were generally correlated with increased termination and decreased pause times, whereas fast elongation was associated with decreased termination. The possibility that poly(A) site recognition and cleavage might also be influenced by elongation speed and/or pause duration is consistent with current knowledge of the mechanisms of these processes. Indeed, pausing downstream of the poly(A) site has been suggested to be important for both polyadenylation and subsequent Pol II termination (Gromak *et al.* 2006).

Overall polymerization rate and/or pausing are thought to contribute to termination by several mechanisms, some of which could be envisioned also to influence the efficiency of poly(A) site recognition and RNA cleavage. In prokaryotic systems, both the response to RNA sequence elements and interactions with accessory proteins are facilitated by polymerase pausing at strategic locations (reviewed in Landick 2006). In eukaryotic cells, the binding of 3′ end processing components to the Pol II CTD facilitates the interaction of these proteins with the poly(A) site as it emerges from the RNA exit tunnel (Kuehner *et al.* 2011). Elongation rate would determine both the length of time the relevant RNA sequences are in close proximity to the polymerase and also the relative timing of synthesis of the separated blocks of RNA sequence needed for assembly of the complete poly(A) processing complex. This sort of kinetic coupling contributes to the efficiency of splicing and the selection of alternative splice sites (Muñoz *et al.* 2010). Changes in elongation rate can also change the pattern of gene expression (Ip *et al.* 2011), which in turn could influence the synthesis and availability of elongation, termination, and processing proteins.

Our initial characterization *in vitro* of Pol II variants mutated in the fork domain is consistent with the hypothesis that faster elongation speed can contribute to greater readthrough (C. E. Kubicek and D. K. Hawley, unpublished data). However, the relationship may be more complicated than that simple correlation suggests because we have observed that mutations in other Pol II domains that also affect elongation rate *in vitro* do not always show the expected readthrough phenotype. The variety of observed behaviors suggest that this collection of mutants will be a valuable resource for dissecting the mechanistic relationships between elongation rate, pausing, termination, and RNA processing events.

The finding that numerous lobe mutations were identified in our study as well as in termination screens of bacterial RNAP and yeast Pol III (Landick *et al.* 1990, Shaaban *et al.* 1995) was initially somewhat surprising. Unlike the fork domain or the other highly conserved residues mutated in our screen, the sequence of the lobe domain is not universally conserved, with the exception of homology region C, which was not represented by a single mutation in our screen. Phenotypes associated with lobe mutations in bacteria have implied a role for that domain in establishing and maintaining the elongation bubble (*e.g.*, Bartlett *et al.* 1998, Trautinger and Lloyd 2002), leading Trinh *et al*. to propose that the increased termination associated with some lobe mutations may reflect an increased propensity for the elongation bubble to collapse at the terminator (Trinh *et al.* 2006).

For both Pol II and Pol III, the termination mutants in the lobe may reflect an altered interaction with another protein. TFIIF is a candidate for that protein in the Pol II system. This conclusion is based on the preponderance of mutations that map to the previously identified TFIIF binding surface and the similar phenotypes of mutants shown to have altered interactions with TFIIF. TFIIF stimulates transcription elongation *in vitro* and has been assumed also to do so *in vivo*, although it has been difficult to verify association of TFIIF with active Pol II elongation complexes in yeast (Krogan *et al.* 2002, Pokholok *et al.* 2002, Mayer *et al.* 2010, Rhee and Pugh 2012).

Recent work in the Pol III system may provide precedent for the hypothesis that TFIIF—or possibly another protein that interacts with the same Pol II surface—has a role in Pol II termination. A subcomplex of two polypeptides considered to be integral Pol III subunits, Rpc37/53, has been proposed to be the Pol III-specific paralog of TFIIF (Kuhn *et al.* 2007). Based on crosslinking experiments, Rpc37/53 associates with the lobe and external 2 domains of Ret1 (Wu *et al.* 2011) and contributes to termination (Landrieux *et al.* 2006). Interestingly, Rpc37/53 and TFIIF might be expected to elicit opposite effects because the intact Pol III is slower, exhibits longer-duration pausing, and terminates more efficiently than the enzyme lacking Rpc37/53 (Landrieux *et al.* 2006), whereas TFIIF has been shown to increase Pol II elongation rate and decrease pausing (reviewed in Shilatifard *et al.* 2003). All but one of the Ret1 lobe mutants with strong termination phenotypes increased readthrough (Shaaban *et al.* 1995). One of these Pol III variants was selected for further study and shown to have a faster elongation rate and reduced propensity for pausing *in vitro* (Shaaban *et al.* 1996), consistent with expectations if the mutation caused a decreased association with Rpc37/53. In contrast, the lobe mutations in our study were found in decreased readthrough strains, which, by analogy, is the phenotype expected if the Pol II mutations disturbed the functional interaction with TFIIF.

Many of the surface substitutions in the protrusion and external 2 domains also altered residues corresponding to or next to positions found to crosslink to TFIIF (Figure 6B). Unlike the lobe mutations, the large majority of these mutations conferred a decreased readthrough phenotype. One possible explanation to reconcile these observations is that the TFIIF contacts may differ in elongation complexes and preinitiation complexes (PICs). For example, some protrusion domain contacts observed for the PIC were absent from the isolated Pol-TFIIF complex (Eichner *et al.* 2010). Interference with normal protrusion/external 2 domain contacts might impair a function of TFIIF that uniquely occurs at or shortly after initiation, whereas the lobe mutant phenotypes may reflect a downstream function, such as elongation speed and pausing in the vicinity of the poly(A) or termination site. Alternatively, during elongation other proteins may associate with surfaces contacted by TFIIF at the promoter. The *rpb2* mutants described here provide a unique tool for answering these and other questions about the contributions of Pol II and associated proteins to polyadenylation and termination.

## Supplementary Material

Supporting Information
