# Supplementary material for: RNA Polymerase II Mutations Conferring Defects in Poly(A) Site Cleavage and Termination in Saccharomyces cerevisiae
Source: G3 (Bethesda). 2013 Feb 1;3(2):167–80. doi: 10.1534/g3.112.004531 (PMC3564978; doi:10.1534/g3.112.004531)
Supplement: Supporting Information [file supp_3_2_167__index.html]

Supporting Information 

# RNA Polymerase II Mutations Conferring Defects in Poly(A) Site Cleavage and Termination in *Saccharomyces cerevisiae*

## Supporting Information for Kubicek *et al.*, 2013

**Files in this Data Supplement:**

- Supporting Information - Figure S1, Table S1, and File S1 (PDF, 208 KB)
- Figure S1 - Analysis of readthrough at the *ADH2* locus using specifically primed cDNAs (PDF, 172 KB)
- Table S1 - Primers used in this study (PDF, 72 KB)
- File S1 - qRT-PCR dataset (.xlsx, 66 KB)
