# Supplementary material for: RNA Polymerase II Mutations Conferring Defects in Poly(A) Site Cleavage and Termination in Saccharomyces cerevisiae
Source: G3 (Bethesda). 2013 Feb 1;3(2):167–80. doi: 10.1534/g3.112.004531 (PMC3564978; doi:10.1534/g3.112.004531)
Supplement: Supporting Information [file supp_3.2.167_004531SI.pdf]

G3· Genes | Genomes | Genetics

RNA Polymerase II Mutations Conferring Defects in Poly(A) Site Cleavage and Termination in *Saccharomyces cerevisiae*

Charles E. Kubicek *, Robert D. Chisholm *, Sachiko Takayama, and Diane K. Hawley

Department of Chemistry and Institute of Molecular Biology, University of Oregon, Eugene, OR 97403-1229

DOI: 10.1534/g3.112.004531

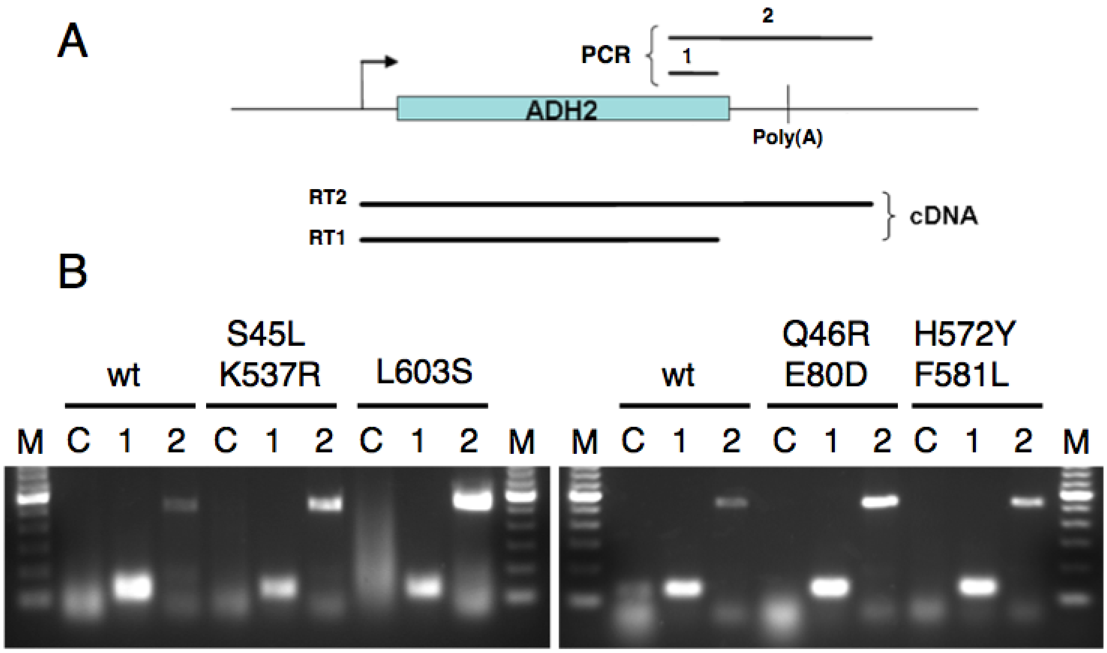

Figure S1 Analysis of readthrough at the *ADH2* locus using specifically primed cDNAs**.** (A) A schematic view of the *ADH2* locus and the expected products of cDNA synthesis and subsequent PCR analysis are shown. Total RNA isolated from yeast strains with mutant *rpb2* alleles was used to synthesize two cDNAs in the same reaction, as described in Materials and Methods. RT1 was synthesized using primer BC118 (Table S1) and RT2, using BC133. The cDNAs were then amplified in separate reactions to obtain PCR products 1 and 2. The primers used for the PCR reactions, which were the same as in the experiment of Figure 3, are listed in Table S1. (B) The products of PCR amplication reactions 1 and 2 were electrophoresed on an agarose gel for the wild-type and indicated *rpb2* strains. The control reaction (C) was a PCR amplification of a cDNA synthesis mock reaction that lacked reverse transcriptase. DNA size markers are also shown (M).

File S1

qRT-PCR dataset

File S1 is available for download at http://www.g3journal.org/lookup/suppl/doi:10.1534/g3.112.004531/-/DC1.

Table S1 Primers used in this study

Name	Sequence	Direction	Gene/position relative to ATG	Purpose
DHO86	CTGCTTGGCGTTCAAAATG	forward	*RPB2*/-401 to -383	PCR mutagenesis
Rpb2xbr	GCAGGATCAACATCGAGATC	reverse	*RPB2*/+2158 to +2177	PCR mutagenesis
BC117	CAAGTCTATCTCCATTGTCGG	forward	*ADH2*/+858 to +878	RT-PCR
BC116	CCGTTTCATCATTGAACTTCG	reverse	*ADH2*/+1377 to +1397	RT-PCR
BC130	TCTGGTAAACTGGATAAGCCA	reverse	*ADH2*/+963 to +983	RT-PCR
BC118	GAAGTGTCAACAACGTATCTACC	reverse	*ADH2*/+1018 to +1040	cDNA synthesis
BC133	CTGAGAAACTATATGAGGGTG	reverse	*ADH2*/+1536 to +1556	cDNA synthesis
DHO17	GTTAGCGCAGTCGTTAAGGC	forward	*ADH2*/+685 to +704	qRT-PCR of *ADH2* ORF
DHO18	AGACAACAGTACCGTTCGC	reverse	*ADH2*/+784 to +802	qRT-PCR of *ADH2* ORF
DHO9	GCATCTTTAGATGACAGTGTTC	forward	*ADH2*/+1112 to +1133	qRT-PCR of *ADH2* poly(A) site
DHO10	GAATGGGTACAACTCACAGG	reverse	*ADH2*/+1212 to +1231	qRT-PCR of *ADH2* poly(A) site
DHO13	GTCCTGCTCCTCTGAATCG	forward	*ADH2*/+1309 to +1327	qRT-PCR of *ADH2* downstream sequence
DHO14	GAGATGAGATGAGTAAATGACA	reverse	*ADH2*/+1407 to +1428	qRT-PCR of *ADH2* downstream sequence
