# Supplementary material for: RNA Polymerase II Mutations Conferring Defects in Poly(A) Site Cleavage and Termination in Saccharomyces cerevisiae
Source: G3 (Bethesda). 2013 Feb 1;3(2):167–80. doi: 10.1534/g3.112.004531 (PMC3564978; doi:10.1534/g3.112.004531)
Supplement: Supporting Information [file supp_3.2.167_FigureS1.pdf]

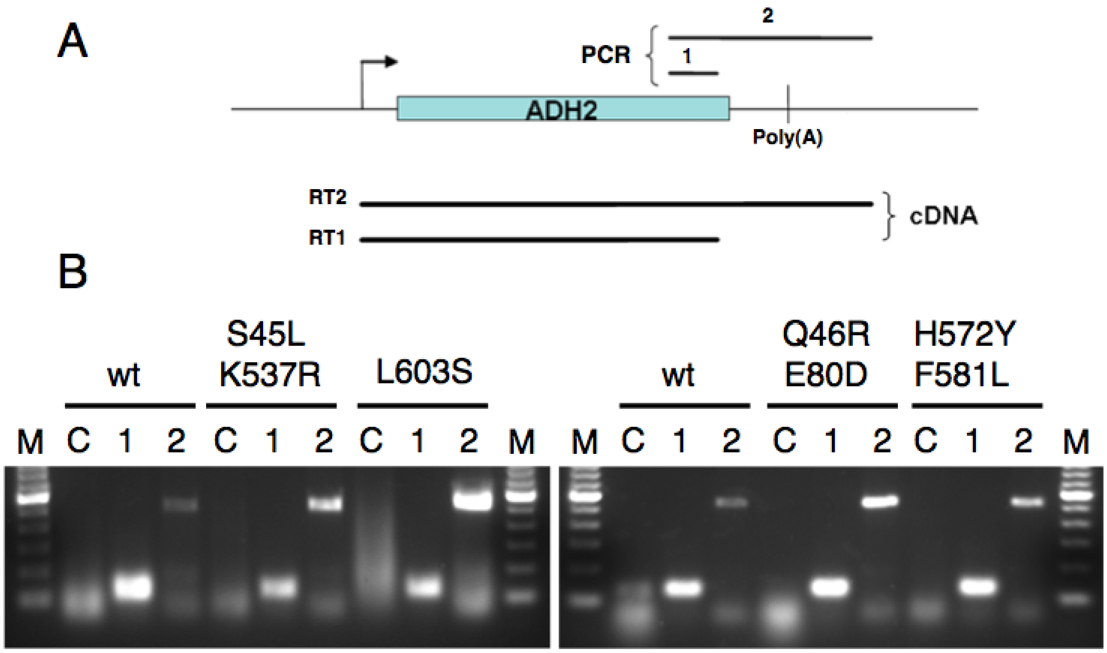

Figure S1 Analysis of readthrough at the *ADH2* locus using specifically primed cDNAs**.** (A) A schematic view of the *ADH2* locus and the expected products of cDNA synthesis and subsequent PCR analysis are shown. Total RNA isolated from yeast strains with mutant *rpb2* alleles was used to synthesize two cDNAs in the same reaction, as described in Materials and Methods. RT1 was synthesized using primer BC118 (Table S1) and RT2, using BC133. The cDNAs were then amplified in separate reactions to obtain PCR products 1 and 2. The primers used for the PCR reactions, which were the same as in the experiment of Figure 3, are listed in Table S1. (B) The products of PCR amplication reactions 1 and 2 were electrophoresed on an agarose gel for the wild-type and indicated *rpb2* strains. The control reaction (C) was a PCR amplification of a cDNA synthesis mock reaction that lacked reverse transcriptase. DNA size markers are also shown (M).
