# Supplementary material for: RNA Polymerase II Mutations Conferring Defects in Poly(A) Site Cleavage and Termination in Saccharomyces cerevisiae
Source: G3 (Bethesda). 2013 Feb 1;3(2):167–80. doi: 10.1534/g3.112.004531 (PMC3564978; doi:10.1534/g3.112.004531)
Supplement: Supporting Information [file supp_3.2.167_TableS1.pdf]

Table S1 Primers used in this study

Name	Sequence	Direction	Gene/position relative to ATG	Purpose
DHO86	CTGCTTGGCGTTCAAAATG	forward	*RPB2*/-401 to -383	PCR mutagenesis
Rpb2xbr	GCAGGATCAACATCGAGATC	reverse	*RPB2*/+2158 to +2177	PCR mutagenesis
BC117	CAAGTCTATCTCCATTGTCGG	forward	*ADH2*/+858 to +878	RT-PCR
BC116	CCGTTTCATCATTGAACTTCG	reverse	*ADH2*/+1377 to +1397	RT-PCR
BC130	TCTGGTAAACTGGATAAGCCA	reverse	*ADH2*/+963 to +983	RT-PCR
BC118	GAAGTGTCAACAACGTATCTACC	reverse	*ADH2*/+1018 to +1040	cDNA synthesis
BC133	CTGAGAAACTATATGAGGGTG	reverse	*ADH2*/+1536 to +1556	cDNA synthesis
DHO17	GTTAGCGCAGTCGTTAAGGC	forward	*ADH2*/+685 to +704	qRT-PCR of *ADH2* ORF
DHO18	AGACAACAGTACCGTTCGC	reverse	*ADH2*/+784 to +802	qRT-PCR of *ADH2* ORF
DHO9	GCATCTTTAGATGACAGTGTTC	forward	*ADH2*/+1112 to +1133	qRT-PCR of *ADH2* poly(A) site
DHO10	GAATGGGTACAACTCACAGG	reverse	*ADH2*/+1212 to +1231	qRT-PCR of *ADH2* poly(A) site
DHO13	GTCCTGCTCCTCTGAATCG	forward	*ADH2*/+1309 to +1327	qRT-PCR of *ADH2* downstream sequence
DHO14	GAGATGAGATGAGTAAATGACA	reverse	*ADH2*/+1407 to +1428	qRT-PCR of *ADH2* downstream sequence
